# High EZH2 Protein Expression Is a Poor Prognostic Predictor in IDH1 R132H-Negative Gliomas

**DOI:** 10.3390/diagnostics12102383

**Published:** 2022-09-30

**Authors:** Yin Ping Wong, Roziasyazni Che Abdul Aziz, Azimatun Noor Aizuddin, Muhamad Fakhri Mohd Saleh, Roslina Mohd Arshad, Geok Chin Tan

**Affiliations:** 1 Department of Pathology, Faculty of Medicine, Universiti Kebangsaan Malaysia, Jalan Yaacob Latif, Bandar Tun Razak, Kuala Lumpur 56000, Malaysia; 2 Department of Community Health, Faculty of Medicine, Universiti Kebangsaan Malaysia, Jalan Yaacob Latif, Bandar Tun Razak, Kuala Lumpur 56000, Malaysia

**Keywords:** EZH2, glioma, IDH1 R132H, immunohistochemistry, overall survival, prognosis, progression-free survival

## Abstract

Accumulating data indicates that enhancer of zeste homology 2 (EZH2) and isocitrate dehydrogenase 1 (IDH1) are implicated in promoting tumourigenesis in a myriad of malignancies including gliomas. We aimed to determine the immunoexpression of EZH2 in gliomas and its correlation with clinicopathological variables. The prognostic value of the combined immunoexpression of EZH2 and IDH1 was further explored in a retrospective analysis involving 56 patients with histologically confirmed gliomas in Universiti Kebangsaan Malaysia Medical Centre from 2010 to 2016. The patients were then followed up for a period of five years. EZH2 and IDH1 R132H immunoexpressions were performed and analysed on respective tissue blocks. Five-year progression-free survival (PFS) and overall survival (OS) were estimated by Kaplan–Meier analysis. Univariate and multivariate Cox proportional hazard regression models were performed to evaluate the value of EZH2 as an independent factor for the prediction of PFS and OS. High EZH2 immunoexpression was demonstrated in 27 (48.2%) gliomas. High EZH2 expression was significantly correlated with older age (*p* = 0.003), higher tumour grade (*p* < 0.001), negative IDH1 R132H immunoexpression (*p* = 0.039), a poor 5-year PFS (mean = 9.7 months, *p* < 0.001) and 5-year OS (mean = 28.2 months, *p* = 0.007). In IDH1 R132H-negative gliomas, there was a trend toward shorter 5-year PFS (mean = 8.0 months, *p* = 0.001) and 5-year OS (mean = 28.7 months, *p* = 0.06) in gliomas demonstrating high EZH2 expression compared with those with low EZH2 expression. High EZH2 immunoexpression is an unfavourable independent prognostic predictor of poor survival in gliomas. EZH2 analysis might therefore be of clinical value for risk stratification, especially in patients with IDH1 R132H-negative gliomas.

## 1. Introduction

Gliomas represent the most commonly occurring primary intracranial tumour in adults, accounting for 31% of all brain and central nervous system (CNS) tumours [1]. The overall age-adjusted incidence rates for all types of gliomas range from 4.67 to 5.73 per 100,000 people [2]. Although brain and CNS tumours are relatively rare compared with other malignancies such as breast and prostate, they are responsible for a disproportionate burden of disease morbidity and mortality, with a high fatality rate (33% overall survival at 5 years after diagnosis) [3].

Current treatment options for gliomas are multimodal, encompass maximal safe surgical resection, radiation therapy and chemotherapy. Despite recent advances in the treatment of the disease, the median survival of patients with gliomas remains dismal, especially for those with invasive and malignant gliomas. The highly infiltrative nature of these tumours makes total surgical resection almost impossible, conferring a high recurrence rate and early disease progression [4]. Thus, there is an urgent need to identify potential prognostic biomarkers of the disease to help predict the clinical outcomes of high-risk patient groups and to tailor therapeutic regimens to improve their quality of life and survival time.

Enhancer of zeste homology 2 (EZH2) has obtained increasing attention in recent years in the field of cancer therapy. It is found to be aberrantly overexpressed in a myriad of malignant tumours including breast, lung, prostate, and stomach malignancies [5]. EZH2 is the catalytic subunit of polycomb repressive complexes 2 [6], which mediates trimethylation of histone H3 lysine 27 (H3K27me3). It is believed to play a critical role in the epigenetic silencing of tumour-suppressor genes and is involved in various biological functions such as cell proliferation and cell differentiation. EZH2 alterations (gain or loss of function mutations) could perturb physiological epigenetic programmes—a pivotal driver to promote tumourigenesis [7].

The overexpression of EZH2 is implicated in aggressiveness and poor prognosis in a wide spectrum of malignancies including cancers of the prostate, breast, female genital tracts, and melanoma. Likewise, in gliomas, EZH2 aberrant expression is a predictor of poor prognosis. EZH2 is believed to play a tumourigenic role in gliomas by epigenetic modulation through methylated histones and the activation of downstream transcription molecules including STAT3 and GATA4 [8]. Several previous studies investigated the prognostic role of EZH2 in glioma patients [9,10,11]; however, the results remained controversial. For instance, Wu et al. (2013) revealed that EZH2 overexpression is a poor prognostic marker for overall survival in patients with gliomas following surgical resection [9]. Nonetheless, Ailon et al. (2015) failed to demonstrate any association between EZH2 immunoexpression and OS in glioma patients [10].

Isocitrate dehydrogenase 1 (IDH1) is one of the three isoforms of the IDH enzyme that plays significant functions in the metabolic Krebs cycle. IDH1 involves in catalyses the oxidative decarboxylation of isocitrate to alpha-ketoglutarate (α -KG). Mutation in the gene encoding IDH1 could result in the gain of IDH1 function and subsequent de novo production of R-2-hydroxyglutarate (R-2-HG), an oncometabolite. This oncometabolite is thought to promote gliomagenesis by upregulating tumour vasculogenesis and altering the epigenome of glioma cells [12]. It is reported that in more than 70% of WHO grade 2/3 cases and secondary astrocytoma, grade 4, carry a heterozygous missense mutation at the IDH1 codon 132, the substitution of Arg132 with histidine (IDH1-R132H). The presence of the IDH1 mutation in glioma predicts a favourable disease outcome with prolonged median survival [13]. Nonetheless, the incidence, prognostic potential, and risk stratification significance of EZH2 expression in relation to IDH1 R132H protein mutant status and gliomas of various grades has not been fully elucidated.

Previous studies utilised molecular profiling as tools to investigate the role of alterations in EZH2 expression in glioma progression [14,15,16]. Nonetheless, the identification of molecular profiles by DNA sequencing is costly, requires intensive laboratory work by trained personnel using specialised equipment, and is not available for daily clinical use in every pathology laboratory. Conversely, immunohistochemistry methods are cheaper and more widely available. This study aimed to investigate EZH2 immunoexpression in glioma patients and explore its clinical significance in tumour progression in relation to IDH1 R132H protein mutant status. The prognostic value of EZH2 as a tissue protein marker for gliomas was also explored.

## 2. Materials and Methods

### 2.1. Study Design and Recruitment of Study Populations

This was a retrospective, cross-sectional study involving all patients that were histologically diagnosed as having primary gliomas in Universiti Kebangsaan Malaysia Medical Centre from January 2010 to December 2016. The clinicopathological characteristics of these patients were reviewed retrospectively by examining the respective medical reports, histology reports, and histologic materials. Patients who underwent neoadjuvant treatment prior to tissue sampling, cases with equivocal histological features or indefinite diagnosis, or cases with paraffin-embedded tissue blocks that were not available due to being lost, insufficient, or destroyed were excluded from this study. Ethical approval was granted by the Medical Research Ethics Committee of Universiti Kebangsaan Malaysia (JEP-2020-082).

A universal sampling method was used in this study. All respective histological slides were retrieved and reviewed by two histopathologists. The histological diagnosis and tumour grades were classified according to the latest recommendations of the World Health Organization [17]. The sections that best represented the lesions were selected, and their respective formalin-fixed paraffin-embedded (FFPE) tissue blocks were retrieved for immunohistochemical studies using EZH2 and IDH1 R132H antibodies.

### 2.2. Immunohistochemistry Analysis with EZH2 and IDH1 R132H

The tissue blocks were sectioned to approximately 4 µm in thickness and mounted on adhesive glass slides (Platinum Pro White, Product No.: PRO-01, Matsunami Japan, Kanagawa, Japan). The slides were left to be air-dried at room temperature overnight. The tissue slides were then incubated on a hotplate at 60 °C for an hour.

Immunohistochemical staining was performed on full-tissue sections using the protocol from EnVision FLEX Mini Kit, High pH (Code No. K8023, Dako Agilent, Glostrup, Denmark). An initial deparaffinization and pretreatment step was performed in the Decloaking Chamber™ NxGen (Ref. No.: DC2012-220V, Biocare Medical, Pacheco, CA, USA) using the EnVision FLEX Target Retrieval Solution, High pH (Code No. DM828, Dako Agilent, Denmark) with the conditions of temperature 110 °C and time 30 min, followed by cooling at room temperature for 30 min and rinsing with running tap water for 3 min. The slides were subsequently incubated with EnVision FLEX Peroxidase-Blocking Reagent (Code No. DM821, Dako Agilent, Denmark) for 5 min followed by a washing step.

The slides were then incubated with primary antibody for 30 min at room temperature. The antibodies were primary mouse monoclonal antibodies against (1) EZH2 (dilution: 1/150, Cell Marque Corporation, Rocklin, CA, USA) and (2) IDH1 R132H (dilution: 1/100, Hamburg, Germany). They were then followed by incubation with EnVision FLEX/HRP (Code No. DM822, Dako Agilent, Denmark) for 30 min, before being incubated with DAB-containing substrate working solution for another 7 min. The DAB-containing substrate working solution was prepared by diluting the 50× concentrated EnVision FLEX DAB+ Chromogen with Envision FLEX Substrate Buffer (Code No. K8023, Dako Agilent, Denmark). The washing steps between each reagent were carried out using EnVision FLEX Wash Buffer 20× (Code No. K8007, Dako Agilent, Denmark) diluted to a 1× working solution with deionised water. The slides were subsequently counterstained with Hematoxylin 2 (REF 7231, Thermo Scientific, Waltham, MA, USA) for 5 s after the procedures had been completed, followed by dehydration steps with increasing alcohol concentration (80%, 90%, 100%, and 100%) and two-times xylene. Finally, the slides were mounted using CoverSealTM-X xylene-based mounting medium (Cat. No.: FX2176, Cancer Diagnostics, USA).

The immunohistochemistry staining results were evaluated and scored independently by two histopathologists (Y.P.W. and G.C.T.) blinded to histological diagnosis and patient outcomes. Cases with discrepancies were concurrently reviewed using a multiheaded microscope until a consensus score was reached.

### 2.3. IDH1 R132H Immunohistochemistry Evaluation

A total of ten high-power microscopic fields were examined and the percentage of positively stained tumour cells was calculated. Immunoreaction was scored as positive when at least 10% of the tumour cells demonstrated a strong granular cytoplasmic staining for IDH1 R132H [18].

### 2.4. EZH2 Immunohistochemistry Evaluation

EZH2 immunoreactivity was evaluated on the basis of semi-quantitative estimation of the extent of positively stained tumour cells (0–3) and the staining intensity of the tumour nuclei (0–3), as previously described [9]. Briefly, the extent of EZH2-positive tumour cell nuclei was scored as follows: 0 (0%), 1 (1–10% of positive tumour cell nuclei), 2 (11–50% of positive tumour cell nuclei), and 3 (>50% of positive tumour cell nuclei), whereas the nuclei staining intensity was set as: 0 (negative), 1 (weak), 2 (moderate), and 3 (strong). A final immunoreactive score (IRS) was obtained by multiplying the extent and intensity scores. A final score of ≥5 was regarded as EZH2-high expression, and a final score of <5 was interpretated as EZH2-low expression.

### 2.5. Clinical Outcome Assessment

Five-year clinical follow up data were retrieved and reviewed from patients’ medical records. Progression-free survival (PFS) was defined as the duration of time from the date of surgery to the date of documented recurrence, or the latest date when censored. Overall survival (OS) was measured from the date of surgery to the date of death due to glioma, or last date of last follow-up if the patients were still alive.

### 2.6. Statistical Analysis

All data and results were processed and analysed statistically using the Statistical Package for the Social Sciences (SPSS) version 26.0. Categorical data were analysed using the chi-square test, while a Student *t*-test was used to assess continuous data. The probability of survival was estimated using the Kaplan–Meier method and the differences between the curves were assessed using a log-rank test. Cox proportional hazard regression was performed to evaluate the independent contribution of EZH2 expression on survival prediction. Additionally, the value of EZH2 in combination with the IDH1 immunoexpression status as a prognostic factor for survival was further explored. The differences were considered statistically significant when the *p*-value was less than 0.05.

## 3. Results

### 3.1. Clinicopathological Characteristics of the Enrolled Study Population

A total of 56 patients with gliomas were enrolled in this study. There were 36 males and 20 females, and their mean age was 41.48 ± 21.69 years (range, 1–83 years). In terms of ethnicity, the majority were Malay (58.9%), followed by Chinese (28.6%) and Indian (7.1%). Glioblastoma (42.8%), astrocytoma grade 2 (14.3%), and astrocytoma grade 3 (12.5%) were the three commonest histopathological subtypes, with 11 (19.6%) and 26 (46.4%) of the cases classified as grade 3 and grade 4, respectively. No adjuvant chemo- or radiotherapy was given to 28 (50.0%) patients, while approximately one-fifth of the patients received radiotherapy only (19.6%) and combined chemo- and radiotherapy (19.6%), respectively. The clinicopathological characteristics of the study population are summarised in Table 1.

### 3.2. EZH2 and IDH1 R132H Mutant Protein Immunoreactivity in Human Gliomas

**Figure 1:**
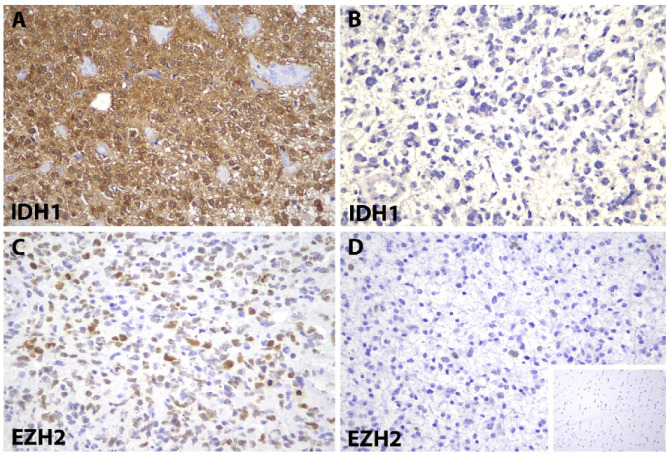
Immunostaining for IDH1 R132H protein mutant and EZH2 in gliomas. (**A**) IDH1 R132H-positive in a case of astrocytoma, grade 4 (IDH1, ×400). (**B**) IDH1 R132H-negative in a case of astrocytoma, grade 3 (IDH1, ×400). (**C**) High EZH2 expression in a case of glioblastoma, grade 4 (EZH2, ×400). (**D**) Low expression of EZH2 in a case of astrocytoma, grade 2. Inset shows EZH2 immunonegativity in adjacent reactive brain tissue (EZH2, ×400). Abbreviations: IDH, isocitrate dehydrogenase; EZH2, enhancer of zeste homologue 2.

Of the 56 cases of gliomas, IDH1 R132H mutant protein expression was seen in the neoplastic glial cells in 16 (28.6%) cases, while the majority (n = 40, 71.4%) were completely negative for the IDH1 R132H mutant protein (Figure 1). EZH2 protein was seen expressed in tumour cell nuclei. High EZH2 protein expression was observed in 27 (48.2%) out of 56 gliomas (Figure 1).

### 3.3. Correlations between EZH2 Protein Expression and Clinicopathological Parameters

The analysed clinicopathological parameters are shown in Table 2 in relation to EZH2 protein expression in the tumour tissue. The high EZH2 expression group was significantly associated with older age group (*p* = 0.003), higher tumour grade (*p* < 0.001), and IDH1 R132H immunonegativity (*p* = 0.039). Patients who were offered combined adjuvant chemo- and radiotherapy were also those diagnosed with at least a grade 3 tumour, and hence, were significantly associated with higher EZH2 protein expression (*p* = 0.003). No significant differences in other host factors, such as the patient’s gender and ethnicity, tumour size, focality, or location, were observed between the high and low EZH2 expression groups.

### 3.4. Prognostic Value of EZH2 in Human Gliomas

A Kaplan–Meier survival curve analysis with a log-rank comparison were proposed to assess disease prognosis. Among the 56 patients that were evaluated, 17 (30.4%) had died and 22 (39.3%) had defaulted along the 5-year follow-up period. As many as 25 cases (44.6%) had recurred during the study period.

**Figure 2:**
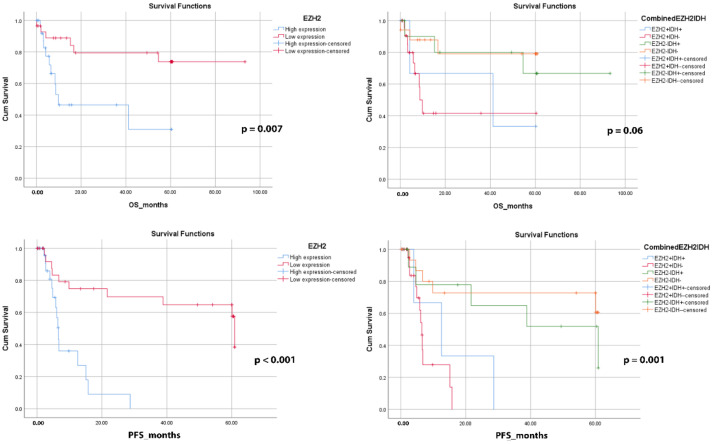
Kaplan–Meier survival curves for 5-year overall survival and 5-year progression-free survival in glioma patients according to EZH2 and combined EZH2/IDH1 immunoexpression status. Abbreviations: PFS, progression-free survival; OS, overall survival.

The high EZH2 expression group was shown to have a significant impact on OS and PFS in patients with gliomas, with a significant decreased OS (mean = 28.2 months, *p* = 0.007) and PFS (mean = 9.7 months, *p* < 0.001) compared to the low EZH2 expression group (mean OS = 73.663 months, *p* = 0.007, and mean PFS = 43.7 months, *p* < 0.001). When combined with IDH1 R132H mutant protein expression status, the high EZH2/IDH1-negative group had significantly affected survival rates. In IDH1 R132H-negative gliomas, there was a trend toward shorter 5-year PFS (mean = 8.0 months, *p* = 0.001) and 5-year OS (mean = 28.7 months, *p* = 0.06) in gliomas demonstrating high EZH2 expression compared with those having low EZH2 expression (Figure 2).

In the univariate analysis, high EZH2 protein expression was a strong independent predictor of poor 5-year PFS in patients with gliomas, with a higher risk of recurrence (hazard ratio of 5.841 with 95% confidence interval 2.231–15.292, *p* < 0.0001). When corrected for other predictive factors including age at diagnosis, tumour grade, tumour focality, and adjuvant therapy status in multivariate analysis, tumours with high EZH2 protein expression were significantly associated with poor 5-year PFS outcome, with a hazard ratio of 6.784 (95% confidence interval 1.465–31.421, *p* = 0.014). Other factors, such as older age at diagnosis, higher tumour grade, and tumour multifocality identified as having strong associations with EZH2 at the univariate level, were not independently associated with a shorter disease-free interval at the multivariable level (Table 3). Additionally, a high expression of EZH2 and IDH1 R132H-negative predicted poor PFS outcome with a higher recurrence risk (hazard ratio of 7.753 with 95% confidence interval 2.321–25.891, *p* = 0.001) at the univariate level; however, this did not meet statistical significance at the multivariable level.

High EZH2 expression served as an independent predictor of poor 5-year OS in gliomas, with a hazard ratio of 3.824 (95% confidence interval 1.357–10.779, *p* = 0.011). Multivariate analysis using the Cox proportional hazard model was performed and included stratification factors such as tumour grade, tumour focality, adjuvant therapy status, and EZH2 immunoexpression. All variables with *p* < 0.05 in univariate analysis were included in the multivariate Cox regression model. High EZH2 expression and high tumour grade were identified as significantly poorer prognostic factors for OS with a higher mortality risk (hazard ratio 6.992, 95% confidence interval 1.317–37.118, *p* = 0.022 and hazard ratio 24.863, 95% confidence interval 3.227–191.544, *p* = 0.002, respectively) (Table 4). Similarly, the high EZH2 expression/IDH1 R132H-negative group predicted a significantly poorer OS outcome with a mortality risk 4.7 times higher than gliomas with low EZH2 expression/IDH1 R132H-negative group at the univariate level (*p* = 0.025), but this was not statistically significant at the multivariable level.

## 4. Discussion

This study provided an overview with regard to the incidence of gliomas in a tertiary referral centre in a developing country over a six-year period. Glioblastoma, IDH-wildtype was the commonest histological subtype, accounting for almost 50% of all gliomas. Similar findings were reported in some tertiary hospitals locally [19] and in the United States [20]. There was a slight male preponderance in the incidence of gliomas, with a male-to-female ratio of 1.8 to 1, consistent with other studies [19,20]. The mean age at presentation was 41.48 years, with the incidence of gliomas the highest in the Malay ethnicity. This can be explained by the fact that Malays make up the majority of the Malaysian population.

Our study demonstrated that EZH2 was upregulated in human gliomas of WHO grades 1 to 4, with an incidence of EZH2 positivity of 48.2%. Pyo et al. (2017), in their meta-analysis, revealed that the incidence of EZH2 positivity could range from 47.2% to 94.7%. The variability of the EZH2 incidence could be related to the inclusion of tumours with variable grades, different EZH2 cut-off, or detection methods [21]. Our analysis observed that high EZH2 expression was significantly associated with higher tumour grades, with EZH2-positive rates of 3.7%, 29.6%, and 66.7% in WHO grade 2, 3, and 4 gliomas, respectively. None of the WHO grade 1 gliomas exhibited high EZH2 immunoexpression. High EZH2 expression was found to be a helpful indicator for supporting the diagnosis of a high-grade glioma. Our results were in line with previous studies [9,11,20], in which the EZH2 positivity rate increased with the increasing WHO tumour grades. This further supported the previous claim that EZH2 plays a pivotal role in glioma development and progression. Low EZH2 immunoexpression was also observed in lower-grade gliomas (WHO grade 1 and 2), while non-neoplastic adjacent brain tissue showed complete EZH2 immunonegativity. Therefore, the potential diagnostic application of EZH2 in distinguishing gliomas from reactive gliosis could be further explored.

Besides tumour grade, our results verified that EZH2 immunoexpression was significantly associated with other clinicopathological parameters, such as age and IDH1 R132H protein mutant status. Ahmad et al. (2016) revealed that high EZH2 expression was greatly linked to older age, larger tumour size, and lower Karnofsky performance status score [11]. A similar association was also observed in other studies, although was not proven to be statistically significant [9,16,22]. The inconsistent results may reflect differences in the glioma subtypes and variations in the studied age groups.

Several studies investigated the prognostic significance of EZH2 in a myriad of cancer types. Gao et al. (2021) and Melling et al. (2015) showed that EZH2 predicted poor prognosis and accelerated tumour progression in triple-negative breast cancer and prostate cancer, respectively [23,24]. Bae et al. (2022) found that EZH2 had a positive association with nodal metastasis and higher alpha-fetoprotein levels in hepatocellular cancer [25]. Our results verified that high EZH2 immunoexpression was correlated with worse 5-year OS and PFS in gliomas, in line with previously published studies [9,16,21,25,26,27]. A meta-analysis performed by Zhang et al. (2017) revealed that EZH2 was predictive for poor 5-year OS and PFS in glioma patients, especially in Asian patients, using the immunohistochemistry method [27]. Similarly, Pyo et al. (2017), in their meta-analysis involving 12 eligible studies, concluded that EZH2 positivity (by immunohistochemistry or polymerase chain reaction) was significantly correlated with WHO tumour grade and conferred a worse prognosis in patients with gliomas [21].

Many studies have been previously performed to link IDH mutations with known genes such as *CDKN2A*, *PTEN, EGFR*, *PDL1*, and *TP53* in gliomas [28,29]. In the current study, we described the correlation of EZH2 with IDH1 R132H protein mutant status in various grades of gliomas. EZH2 was found to be negatively correlated with IDH1 R132H protein expression, in which EZH2 was highly expressed in IDH1 R132H-negative gliomas (*p* = 0.039). Both EZH2 and IDH1 are known epigenetic modifiers that contribute to the “stemness” of cancer through aberrant histone and DNA methylation, leading to the occurrence and progression of malignancies. While *IDH* mutation is associated with the initiation of glioma, interestingly, *IDH*-mutant gliomas show favourable patient outcomes compared to the wildtype *IDH* counterpart [30]. However, exactly how EZH2 is associated with IDH1 R132H protein mutation status remains largely unknown, pending future investigation.

Next, we evaluated the potential consequence of the IDH1 R132H mutant protein expression in addition to EZH2 and its impact on the patients’ outcomes. To be best of our knowledge, combined EZH2 and IDH1 R132H protein expressions as possible prognostic indicators were not previously studied. Our study revealed that in IDH1 R132H-negative gliomas, EZH2 overexpression predicted significantly poorer PFS and OS outcomes with a higher recurrence risk (*p* = 0.001) and 4.7 times higher mortality (*p* = 0.025) than IDH1 R132H-negative gliomas with low EZH2 expression. However, these results did not meet statistical significance at the multivariable level. A more accurate result is limited due to the small sample size and retrospective nature of data acquisition in the present study.

## 5. Conclusions

In conclusion, EZH2 is highly expressed in higher grade gliomas. It serves as an unfavourable independent prognostic predictor of poor outcomes in gliomas. EZH2 analysis might, therefore, be of clinical value for risk stratification, especially in patients with IDH1 R132H-negative gliomas.

## Figures and Tables

**Table 1:** Clinicopathological parameters of the study population.

Clinicopathological Parameters		Total Number of Cases (%)
Age, years (mean ± SD)		41.48 ± 21.69
Gender	Male	36 (64.3)
	Female	20 (35.7)
Race	Malay	33 (58.9)
	Chinese	16 (28.6)
	Indian	4 (7.1)
	Other	3 (5.4)
Tumour location	Supratentorial	51 (91.1)
	Infratentorial	5 (8.9)
Tumour size, cm (mean ± SD)		4.55 ± 1.78
Histological diagnosis	Pilocytic astrocytoma	5 (8.9)
	Desmoplastic infantile astrocytoma	1 (1.8)
	Astrocytoma, grade 2	8 (14.3)
	Astrocytoma, grade 3	7 (12.5)
	Astrocytoma, grade 4	2 (3.6)
	Glioblastoma, IDH-wildtype, grade 4	24 (42.8)
	Ependymoma, grade 2	2 (3.6)
	Oligodendroglioma, grade 2	3 (5.4)
	Oligodendroglioma, grade 3	4 (7.1)
Tumour grade	Grade 1	6 (10.7)
	Grade 2	13 (23.2)
	Grade 3	11 (19.6)
	Grade 4	26 (46.4)
IDH1 R132H immunoexpression	Positive	16 (28.6)
	Negative	40 (71.4)
EZH2 immunoexpression	High expression	27 (48.2)
	Low expression	29 (51.8)
Combined EZH2/IDH1 R132H	EZH2^H^/IDH1+	4 (7.1)
	EZH2^H^/IDH1−	23 (41.1)
	EZH2^L^/IDH1+	12 (21.4)
	EZH2^L^/IDH1−	17 (30.4)
Adjuvant therapy	No	28 (50.0)
	Radiotherapy only	11 (19.6)
	Combined chemotherapy and radiotherapy	11 (19.6)
	Unknown	6 (10.8)

Abbreviations: EZH2H—high EZH2 expression; EZH2L—low EZH2 expression; + positive; − negative.

**Table 2:** EZH2 immunoexpression in gliomas and its association with clinicopathological parameters.

Clinicopathological Parameters	EZH2 Immunoexpression		*p*-Value
	High Expression (n, %)	Low Expression (n, %)	
Age, years (mean ± SD)	50.3 (±19.3)	33.3 (±20.8)	0.003 *
Gender			0.579
Male	16 (44.4)	20 (55.6)	
Female	11 (55.0)	9 (45.0)	
Race			0.358
Malay	14 (42.4)	19 (57.6)	
Chinese	8 (50.0)	8 (50.0)	
Indian	2 (50.0)	2 (50.0)	
Other	3 (100.0)	0 (0.0)	
Tumour location			0.052
Supratentorial	27 (52.9)	24 (47.1)	
Infratentorial	0 (0.0)	5 (100.0)	
Tumour size, cm (mean ± SD)	4.54 (±1.51)	4.57 (±2.03)	0.953
Tumour focality			0.497
Single	21 (45.7)	25 (54.3)	
Multifocal	6 (60.0)	4 (40.0)	
Tumour grade			<0.001 *
Grade 1	0 (0.0)	6 (100.0)	
Grade 2	1 (7.7)	12 (92.3)	
Grade 3	8 (72.7)	3 (27.3)	
Grade 4	18 (69.2)	8 (30.8)	
Histological diagnosis			<0.001 *
Pilocytic astrocytoma	0 (0.0)	5 (100.0)	
Desmoplastic infantile astrocytoma	0 (0.0)	1 (100.0)	
Astrocytoma, grade 2	0 (0.0)	8 (100.0)	
Astrocytoma, grade 3	7 (100.0)	0 (0.0)	
Astrocytoma, grade 4	1 (50.0)	1 (50.0)	
Glioblastoma, IDH-wildtype, grade 4	17 (70.8)	7 (29.2)	
Ependymoma, grade 2	1 (50.0)	1 (50.0)	
Oligodendroglioma, grade 2	0 (0.0)	3 (100.0)	
Oligodendroglioma, grade 3	1 (25.0)	3 (75.0)	
IDH1 R132H immunoexpression			0.039 *
Positive	4 (25.0)	12 (75.0)	
Negative	23 (57.5)	17 (42.5)	
Adjuvant therapy			0.003 *
No	7 (25.0)	21 (75.0)	
Radiotherapy only	9 (81.8)	2 (18.2)	
Combined chemo- and radiotherapy	7 (63.6)	4 (36.4)	
Unknown	4 (66.7)	2 (33.3)	

* Statistically significant.

**Table 3:** Cox regression models for progression-free survival in patients with gliomas.

Clinicopathological Parameters	Univariate			Multivariate		
	HR	95% CI	*p*-Value	HR	95% CI	*p*-Value
Age						
0–20	1.00			1.00		
21–40	9.995	1.262–79.177	0.029 *	4.176	0.358–48.740	0.254
41–60	12.957	1.643–102.182	0.015 *	5.282	0.442–63.049	0.188
More than 60	12.714	1.455–111.086	0.021 *	4.062	0.218–75.619	0.347
Tumour grade						
Low grade	1.00			1.00		
High grade	6.876	2.479–19.073	<0.001 *	7.081	0.643–77.985	0.110
Location				Not included		
Infratentorial	1.00					
Supratentorial	30.175	0.414–2200.337	0.120			
Focality						
Single	1.00			1.00		
Multiple	4.934	1.527–15.944	0.008 *	2.349	0.604–9.133	0.218
Size				Not included		
Less than 4 cm	1.00					
More than 4 cm	1.253	0.560–2.801	0.583			
Adjuvant therapy						
No	1.00			1.00		
Radiotherapy only	2.263	0.733–6.981	0.155	0.141	0.025–0.794	0.026 *
Both chemotherapy and radiotherapy	5.178	1.918–13.978	0.001 *	0.407	0.074–2.249	0.303
EZH2						
Low expression	1.00			1.00		
High expression	5.841	2.231–15.292	<0.001 *	6.784	1.465–31.421	0.014 *
IDH1 R132H				Not included		
Positive	1.00					
Negative	1.054	0.687–1.616	0.811			
Combined EZH2/IDH1						
EZH2^H^/IDH1+	5.483	1.240–24.245	0.025	-	-	
EZH2^H^/IDH1−	7.753	2.321–25.891	0.001 *	2.436	0.436–13.607	0.310
EZH2^L^/IDH1+	1.509	0.428–5.313	0.522	0.497	0.099–2.504	0.397
EZH2^L^/IDH1−	1.00			1.00		

Abbreviations: CI—confidence interval; EZH2^H^—high EZH2 expression; EZH2^L^—low EZH2 expression; HR—hazard ratio; + positive; − negative; * statistically significant.

**Table 4:** Cox regression models for overall survival in patients with gliomas.

Clinicopathological Parameters	Univariate			Multivariate		
	HR	95% CI	*p*-Value	HR	95% CI	*p*-Value
Age				Not included		
0–20						
21–40	91,576.249	0.0–2.649E	0.932			
41–60	103,821.439	0.0–3.004E	0.931			
More than 60	185,740.458	0.0–5.377E	0.927			
Tumour grade						
Low grade	1.00			1.00		
High grade	6.100	1.671–22.270	0.006 *	24.863	3.227–191.544	0.002 *
Location				Not included		
Infratentorial	1.00					
Supratentorial	26.549	0.078–9083.097	0.271			
Focality						
Single	1.00			1.00		
Multiple	3.113	0.826–11.728	0.093	1.670	0.385–7.249	0.493
**Size**				Not included		
Less than 4 cm	1.00					
More than 4 cm	2.169	0.760–6.189	0.148			
Adjuvant therapy						
No	1.00			1.00		
Radiotherapy only	0.663	0.140–3.144	0.605	0.049	0.008–0.315	0.001 *
Both chemotherapy and radiotherapy	2.172	0.735–6.419	0.161	0.241	0.057–1.011	0.052
EZH2						
Low expression	1.00			1.00		
High expression	3.824	1.357–10.779	0.011 *	6.992	1.317–37.118	0.022 *
IDH1 R132H				Not included		
Positive	1.00					
Negative	1.227	0.427–3.525	0.704			
Combined EZH2/IDH1						
EZH2^H^/IDH-1+	3.795	0.632–22.775	0.145	-	-	-
EZH2^H^/IDH-1−	4.694	1.210–18.200	0.025 *	4.281	0.568–32.286	0.158
EZH2^L^/IDH-1+	1.402	0.283–6.957	0.679	0.488	0.089–2.690	0.295
EZH2^L^/IDH-1−	1.00			1.00		

Abbreviations: CI—confidence interval; EZH2^H^—high EZH2 expression; EZH2^L^—low EZH2 expression; HR—hazard ratio; + positive; − negative; * statistically significant.

## Data Availability

Not applicable.
